# Relative posture between head and finger determines perceived tactile direction of motion

**DOI:** 10.1038/s41598-020-62327-x

**Published:** 2020-03-26

**Authors:** Yueh-Peng Chen, Chun-I Yeh, Tsung-Chi Lee, Jian-Jia Huang, Yu-Cheng Pei

**Affiliations:** 1 Department of Physical Medicine and Rehabilitation, Chang Gung Memorial Hospital, Taoyuan, Taiwan; 2 Center of Vascularized Tissue Allograft, Chang Gung Memorial Hospital at Linkou, Taoyuan, Taiwan; 3 School of Medicine, Chang Gung University, Taoyuan, Taiwan; 4 Healthy Aging Research Center, Chang Gung University, Taoyuan, Taiwan; 5 Center for Artificial Intelligence in Medicine, Chang Gung Memorial Hospital at Linkou, Taoyuan, Taiwan; 6 Department of Psychology, National Taiwan University Taipei, Taiwan

**Keywords:** Somatosensory system, Human behaviour

## Abstract

The hand explores the environment for obtaining tactile information that can be fruitfully integrated with other functions, such as vision, audition, and movement. In theory, somatosensory signals gathered by the hand are accurately mapped in the world-centered (allocentric) reference frame such that the multi-modal information signals, whether visual-tactile or motor-tactile, are perfectly aligned. However, an accumulating body of evidence indicates that the perceived tactile orientation or direction is inaccurate; yielding a surprisingly large perceptual bias. To investigate such perceptual bias, this study presented tactile motion stimuli to healthy adult participants in a variety of finger and head postures, and requested the participants to report the perceived direction of motion mapped on a video screen placed on the frontoparallel plane in front of the eyes. Experimental results showed that the perceptual bias could be divided into systematic and nonsystematic biases. Systematic bias, defined as the mean difference between the perceived and veridical directions, correlated linearly with the relative posture between the finger and the head. By contrast, nonsystematic bias, defined as minor difference in bias for different stimulus directions, was highly individualized, phase-locked to stimulus orientation presented on the skin. Overall, the present findings on systematic bias indicate that the transformation bias among the reference frames is dominated by the finger-to-head posture. Moreover, the highly individualized nature of nonsystematic bias reflects how information is obtained by the orientation-selective units in the S1 cortex.

## Introduction

A hallmark of hand function is to manipulate objects and acquire tactile information, a process denoted as haptics. Furthermore, human manipulation of objects in a series of haptic process requires an integrated neural representation of the body (body schema) and of the space around the body^1–5^. When touching an object with hands, we perceive tactile motion^6^ that is crucial toward determining the direction^7–11^ and speed^8,12,13^ of the object, as well as for planning subsequent movements^14^. While the tactile motion perceived by the skin of hands is encoded in the somatotopic (skin-centered) reference frame^15,16^, the physical movement of the object is actually represented in the allocentric (or external world-centered) reference frame^17,18^.

Misalignment between the reference frames frequently causes a bias in perceiving tactile motion^11,19^. Therefore, tactile remapping is the outcome of multimodality integration that has been shown to be affected by finger postures^20^, body postures^21^, and transformation of eye- and body-centered reference frames^22,23^, indicating a large-scale transformation and integration involving multiple reference frames^24–27^. For example, perceived visual^28^ and tactile^29^ motion directions can be affected by hand and arm postures^30,31^. Studies on temporal integration suggested that multimodal information was processed by a recurrent scheme among associated cortical areas^32,33^. Importantly, sensorimotor contingency, a systematic co-occurrence of sensory and motor events, can produce a new reference frame to improve motor performance^34,35^. The present study investigated the integration process as tactile motion projects on the visual reference frame while manipulating the upper limb and head postures.

The somatotopic frame must take the body posture into account because the perception of physical environments by the hand cannot be disassociated from certain body postures^36^. In practice, body posture is an integrated result derived from the positions of multiple joints^37,38^, including the shoulder, elbow, hand, and wrist^39–41^. Strong evidence suggests that humans have the ability to estimate the end-point of the body extremities (i.e., the hands) with a high degree of precision through the integration of proprioceptive information^42–44^. In other words, the body posture is well organized into the somatotopic frame. Regarding the cutaneous senses of the hand, previous studies have shown that the perception of tactile orientation by the hand cannot be explained by any single reference frame of the body posture. When performed on the horizontal^45,46^, midsagittal^47^, or frontoparallel plane^48^, the perception of tactile orientation must include information from the angles of multiple joints to be integrated with the cutaneous senses. In other words, tactile perceptual bias may be attributed to the existence of intermediate reference frames that are used for multi-sensory integration^49–51^.

However, perceptual bias may also stem directly from cutaneous senses. In general, cutaneous information must be integrated with information regarding the body posture in order to achieve precise haptic processes. For example, when participants were asked to judge whether a bar presented to one palm was parallel to a second bar presented to the other palm, the participants’ judgment was intermediate between the allocentric and somatotopic reference frames^45,46^. Furthermore, tactile direction and orientation presented to the left index fingerpad yielded a clockwise bias of 20°–25° when the left forearm was positioned in a forward and volar side up posture^52,53^. In other words, it appears that tactile orientation and direction share a common transformation process from an object’s physical condition to an individual’s perception.

The present study aimed to characterize the rules governing the perceptual bias underlying the transformation of reference frames. Tactile motions were presented to the left index fingerpad using a miniature tactile stimulator. A video screen was placed between the eyes of the participant and the stimulator, and the participants used a mouse to report the perceived direction of motion of the stimulus on the screen. This study used a design in which the stimulator, left index fingerpad, center of the video screen, and eyes were perfectly aligned along the posterior-to-anterior axis. In performing the trials, the participants’ head and finger postures were manipulated in a controlled manner in order to investigate the effect of these postures on perceptual bias. Results revealed that the perceptual bias was a linear summation of two different types of bias, namely systematic bias and nonsystematic bias. The systematic bias correlated linearly with the difference between the finger and the head postures for all participants. By contrast, the nonsystematic bias is highly individualized, and has a phase determined mainly by the finger posture. The two co-existing biases may represent the underlying principles governing the transformation of reference frames in the perception of tactile motion.

## Results

### Study design

**Figure 1:**
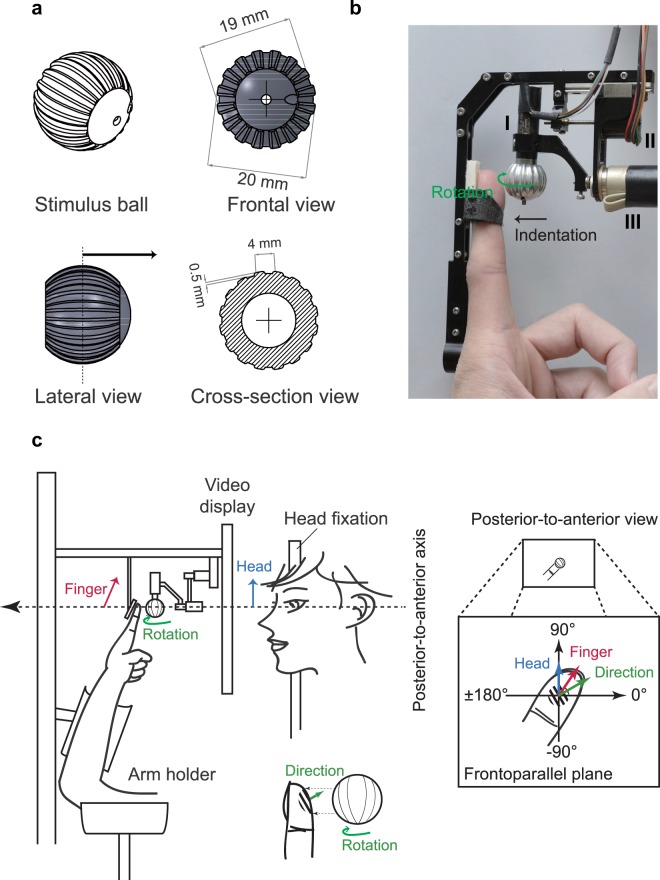
Experimental apparatus and setup. (**a**) The aluminum stimulation ball was engraved with a square-wave grating with a groove depth of 500 μm, a wavelength of 4 mm, and a 45% duty cycle. (**b**) Motion stimulus was delivered to the fingerpad of the left index finger using the miniature tactile stimulator with three motors, namely a DC motor to control the speed of the ball (I); a stepper motor with a spiral shaft to control the vertical indentation depth of the stimulus ball on the skin (II); and a DC motor to control the direction of motion of the ball across the fingerpad (III). (**c**) The participant’s left upper limb was supported by the arm holder with the palm facing the participant’s face such that the left index fingerpad contacted the aluminum ball during stimulation. The participant’s head (blue vector: head orientation), eyes, video display, stimulus (stimulus direction: green vector), and left index fingerpad (finger orientation: red vector) were precisely aligned along the posterior-to-anterior axis. In each trial, the rotating grating ball indented the fingerpad and was driven in a particular direction resulting in tactile stimulation.

This study evaluated the effect of the relative head and finger posture on motion perception at the fingerpad. A rotating aluminum ball with a groove depth of 500 μm, a wavelength of 4 mm, and a 45% duty cycle was used as the tactile stimulus to deliver tactile motions (Fig. 1a). Motion stimulus was delivered to the fingerpad of the left index finger using the miniature tactile stimulator with three motors that can precisely control the speed, direction and indentation depth of the stimulus^54^ (Fig. 1b). Each participant sat in front of a table with the angle of elbow joint kept constant. The participant’s left upper limb was supported by the arm holder with the palm facing the participant’s face such that the left index fingerpad contacted the aluminum ball during stimulation. The participant’s head, eyes, video display, stimulus, and left index fingerpad were precisely aligned along the posterior-to-anterior axis (Fig. 1c, see also Experimental set-up for details).

**Figure 2:**
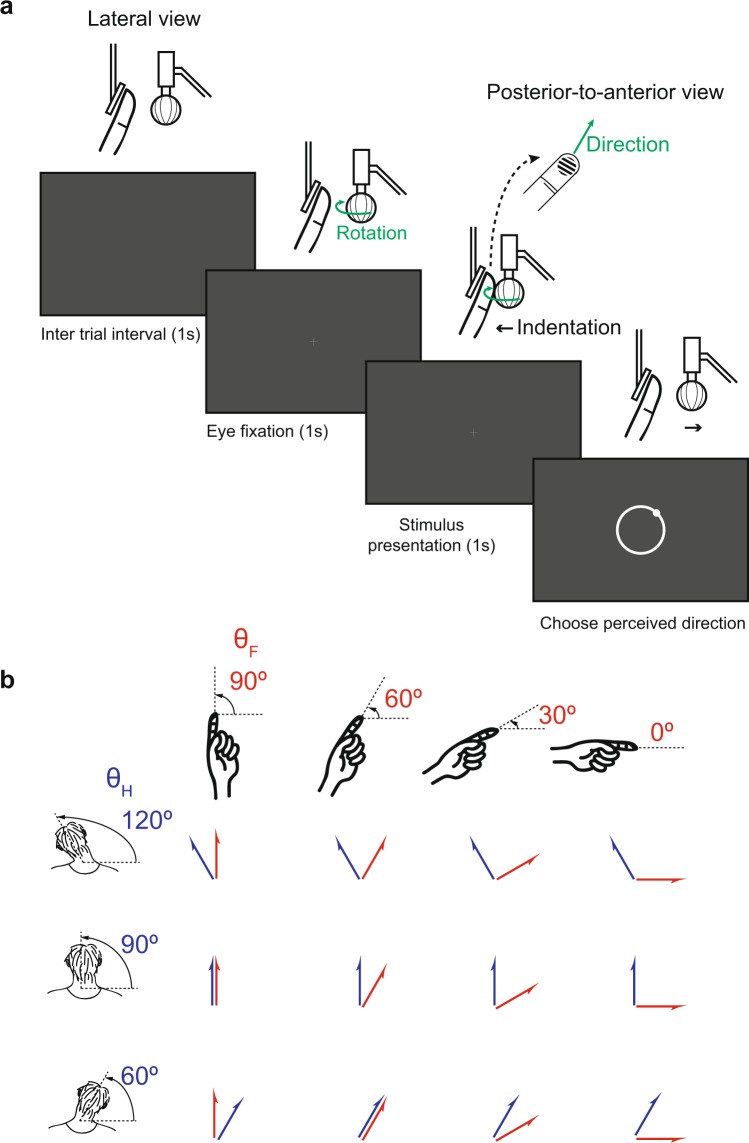
Presentation of tactile stimuli to participants. (**a**) In each trial, the participant visually fixated on a cross symbol displayed at the center of the screen for 1 second. Stimulus motion, chosen from one of 24 different directions, was then presented to the participant’s fingerpad for 1 second. After tactile stimulation, the participant reported the perceived direction of motion by performing a mouse click at a position corresponding to the perceived direction on a circle presented on the screen. The inter-trial interval was set as 1 second. (**b**) The trials were performed using a block design consisting of factorial combinations of three head postures (θ_H_ = [120°, 90°, 60°]) and four finger postures (θ_F_ = [90°, 60°, 30°, 0°]); yielding a total of 12 posture combinations.

The perceived direction of the tactile motion presented to the participant’s left index fingerpad was analyzed for carefully controlled angles of the head and finger. In each trial, the rotating grating ball indented the fingerpad and was driven in a particular direction resulting in tactile stimulation. The participant visually fixated on a cross presented at the center of the video display and reported the perceived direction of motion (${R}_{i}$) using a mouse to click on an appropriate point on a circle shown on the video display (Fig. 2a). The stimulation trials were performed in accordance with a 3-by-4 factorial finger-and-head posture combination design consisting of three head postures and four finger postures. In particular, the finger postures (θ_F_) were set as 90°, 60°, 30° or 0°, while the head postures (θ_H_) were set as 120°, 90° or 60°; yielding a total of 12 different posture combinations (Fig. 2b). The effect of the head and finger postures on the perceived direction of tactile motion was evaluated by comparing the difference between the veridical and perceived directions across all postures.

### Systematic bias

**Figure 3:**
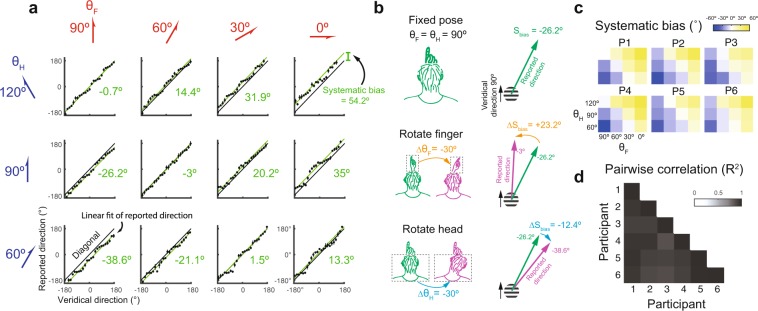
Reported directions of motion and systematic bias. (**a**) Perceived directions of motion as a function of the veridical direction for all the finger (θ_F_) and head (θ_H_) posture combinations in a sample participant. The black diagonal line indicates the prediction where the reported and veridical directions of motion are identical. The green line plots the systematic bias plus veridical direction vs. the veridical direction alone. (**b**) For this participant, given θ_H_ and θ_F_ equal to 90°, the systematic bias had a value of −26.2° (denoted by the green arrow). For a clockwise shift of θ_F_ from 90° to 60° (i.e., Δθ_F_ = −30°, the orange arrow), the systematic bias exhibited a counterclockwise shift from −26.2° to −3° (i.e., ΔS_bias_ = 23.2°, the orange arrow). In other words, a negative correlation exists between the finger posture and the systematic bias. By contrast, a clockwise shift of θ_H_ from 90° to 60° (i.e., Δθ_H_ = −30°, the blue arrow) produced a clockwise shift of the systematic bias from −26.2° to −38.6° (ΔS_bias_ = −12.4°, the blue arrow); indicating the existence of a positive correlation between the head posture and the systematic bias. (**c**) For each participant, the systematic bias was color-coded across the 12 posture combinations. (**d**) Analysis results for pairwise correlations between six participants for systematic bias across finger-head postures are shown in Fig. 3c.

This study aimed to understand how the perceived direction of motion of the stimuli presented to the fingerpad was modulated by the relative posture of the finger and head. According to the data obtained from a single sample participant (Fig. 3a), the systematic bias was found to be modulated by both the head posture (θ_H_) and the finger posture (θ_F_). Specifically, the highest systematic bias occurred for θ_H_ = 120° and θ_F_ = 0°, while the lowest systematic bias occurred for θ_H_ = 60° and θ_F_ = 90°. Interestingly, the systematic bias was approximately zero for postures of θ_H_ = 120° and θ_F_ = 90°, θ_H_ = 90° and θ_F_ = 60°, and θ_H_ = 60° and θ_F_ = 30°, i.e., postures under which the difference between the finger and head angles was −30° (θ_H_ − θ_F_ = −30°). Notably, a clockwise shift of the finger induced a counterclockwise change in the systematic bias, indicating that the finger posture modulated the systematic bias in the opposite direction (Fig. 3b, middle). By contrast, a clockwise shift of the head posture resulted in a clockwise change in the systematic bias. In other words, the head posture modulated the systematic bias in the same direction (Fig. 3b, bottom). These patterns of the systematic bias were found to be similar across all six participants (Fig. 3c), with high values of pairwise correlations of systematic bias across finger-head postures (0.93 ± 0.04, Mean ± SD, across all participants, Fig. 3d).

**Figure 4:**
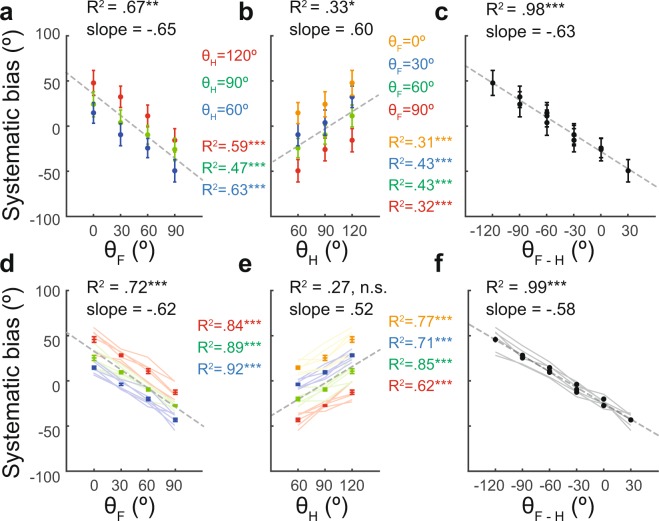
Effects of finger, head and relative finger-head postures on systematic bias. The correlation of systematic bias with three different postures was considered, namely the finger posture (θ_F_), head posture (θ_H_), and relative finger-head posture (θ_F-H_). For the sample participant, systematic bias was linearly modulated by the finger (**a**), head (**b**) and relative finger-head (**c**) postures, respectively; with the relative finger-head posture showing an almost perfect fit. The dashed lines in the figures represent the results of linear regression. (**d-f**) Systematic bias pooled from six participants. In (**d**) and (**e**), each color-coded line represents data from individual participants, and each color represents data from head (θ_H_ = [120°, 90°, 60°] denoted by [red, green, blue]), and finger (θ_F_ = [90°, 60°, 30°, 0°] denoted by [red, green, blue, yellow]). In (**f**), relative finger-head postures (θ_F-H_) of each participant are denoted by gray lines. R^2^ was 0.957 ± 0.029 (all p < 0.001, ranging from 0.92 to 0.99) for the six participants (gray lines). R^2^ = 0.99 is calculated for the systematic bias averaged across all participants. In addition, the error bars show the S.E.M. across the participants. *p  < 0.05, **p < 0.01, ***p < 0.001. Error bars represent S.E.M.

In general, the results presented in Fig. 3 indicate that the systematic bias can be robustly predicted by the head and finger postures. A further analysis was thus performed to clarify the detailed relations of the systematic bias with finger, head and finger-head postures, respectively (Fig. 4a-c for a sample participant, and Fig. 4d-f for all six participants). Results showed that systematic bias was modulated by finger posture in the sample participant (Fig. 4a, for participant #1, slope = −0.65, R^2^ = 0.67, t = −4.53, p = 0.001, df = 10, data from averaged biases for each finger-head posture) and in the mean value across of all six participants (Fig. 4d, slope = −0.62, R^2^ = 0.72, t = −5.1, p < 0.001, df = 10, data from biases averaged across participants). For example, the systematic bias vs. finger posture plot had a slope of −0.65 for the sample participant and close to −0.62 for all participants (Fig. 4a,d); indicating that a change in finger posture induces a change in systematic bias in the opposite direction (see also Fig. 3b, middle). However, systematic bias was significantly modulated by head posture (Fig. 4b, for participant #1, slope = 0.60, R^2^ = 0.33, t = 2.26, p = 0.047, df = 10, data from averaged biases for each finger-head posture) in the sample participant but not in mean value of all participants (Fig. 4e, slope = 0.52, R^2^ = 0.27, t = 1.92, p = 0.083, df = 10, data from biases averaged across participants); indicating that a change in the head posture induces a change of the systematic bias in the same direction in the sample participant (see also Fig. 3b, bottom) but not in all participants.

An investigation was performed to determine the ability of finger posture to predict systematic bias when the head posture was controlled. A good goodness-of-fit (as measured by the R^2^ coefficient) was found for the finger posture in both the sample participant (Fig. 4a, for participant #1, θ_H_ = [120°, 90°, 60°]: slope = [−0.71, −0.55, −0.69], R^2^ = [0.59, 0.47, 0.63], t = [−18.37, −23.65, −25.32], all p < 0.001, df = 382, data from averaged biases for each finger-posture) and all the participants (Fig. 4d, for θ_H_ = [120°, 90°, 60°]: slope = [−0.64, −0.59, −0.63], R^2^ = [0.84, 0.89, 0.92], t = [−10.82, −13.46, −15.65], all p < 0.001, df = 22, data from biases averaged within participants). A further investigation was then conducted to examine the ability of head posture to predict systematic bias where the finger posture was controlled. Results also showed that a good goodness-of-fit for the head posture in both the sample participant (Fig. 4b, θ_F_ = [90°, 60°, 30°, 0°]: slope = [0.55, 0.59, 0.70, 0.55], R^2^ = [0.32, 0.43, 0.43, 0.31], t = [11.58, 14.55, 14.72, 11.32], all p < 0.001, df = 286, data from averaged biases for each finger-head posture) and all the participants (Fig. 4e, for θ_F_ = [90°, 60°, 30°, 0°]: slope = [0.52, 0.54, 0.51, 0.51], R^2^ = [0.62, 0.85, 0.71, 0.77], t = [5.14, 9.68, 6.29, 7.33], all p < 0.001, df = 16, data from biases averaged within participants). A final investigation was performed to examine the degree to which systematic bias could be predicted by the relative finger-head posture (i.e., the difference between finger posture and the head (θ_F-H_)). Results showed almost perfect correlation of systematic bias with finger-head posture (Fig. 4c, for sample participant #1: slope = −0.63, R^2^ = 0.98, t = −22.56, p < 0.001, df = 10, data from averaged biases for each finger-head posture. Figure 4f, for all six participants, slope = −0.58, R^2^ = 0.99, t = −57.18, p < 0.001, df = 4, data from biases averaged across participants); indicating that finger-head posture almost completely determined systematic bias. Finally, the systematic bias vs. finger-head posture plot had a slope of −0.63 for the sample participant and −0.58 across all the participants. In other words, a change in the finger-head posture induced a change of systematic bias in the opposite direction with a similar magnitude across all six participants.

### Nonsystematic bias

**Figure 5:**
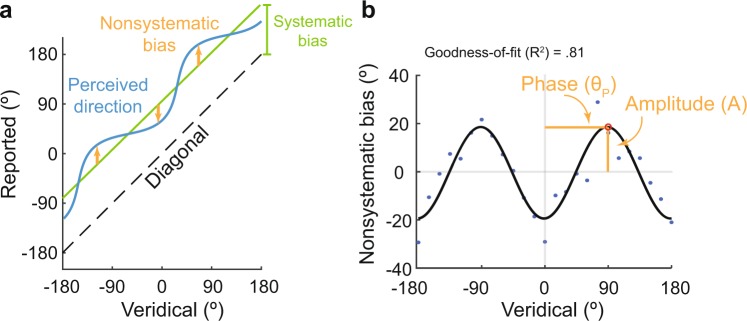
Cosine fit for nonsystematic bias. (**a**) The nonsystematic bias (yellow arrow) varies with the veridical direction of motion. (**b**) A cosine function with moment = 2 provides the best fit of the nonsystematic bias for the sample participant at a posture [θ_H,_ θ_F_] = [60°, 90°]. The peak of the nonsystematic bias can be represented by the amplitude (A) and phase (θ_p_).

In addition to the systematic bias described above, which correlated linearly with the relative posture between the finger and the head, a further bias (denoted as nonsystematic bias) which exhibited different values for different directions of the tactile stimulation was also observed. Nonsystematic bias varied across all stimulus directions (Fig. 5a, blue line); hence, it was fitted with a cosine function. A preliminary investigation found that the best fit was obtained using a cosine function with moment = 2 (Fig. S1a). After the cosine fit was established, two parameters were extracted, namely the amplitude (A) of the nonsystematic bias at its peak position and the corresponding phase (θ_p_) (Fig. 5b). No significant difference was found in the goodness-of-fit across all the finger postures, head postures, and relative finger-head postures (Fig. S1b, one-way repeated-measures ANOVA. For head posture, F(2, 42) = 0.59, p = 0.56. For finger posture, F(3, 45) = 0.63, p = 0.60. For finger - head posture, F(11, 44) = 0.87, p = 0.58).

**Figure 6:**
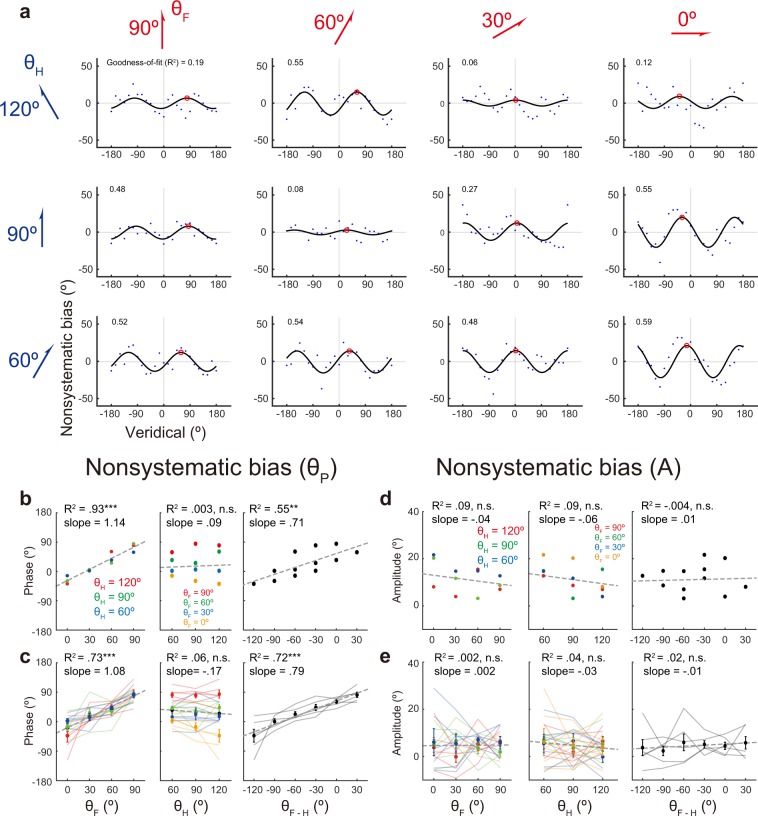
Results of cosine fitting for finger, head and finger-head postures of the sample participant and all six participants. (**a**) For the sample participant, for each posture combination, the nonsystematic bias was fitted with a cosine fit (moment = 2), from which the peak-phase and peak-amplitude (red circles) were then extracted to represent the corresponding nonsystematic bias. (**b**) For the sample participant, the phase of the nonsystematic bias correlated with the two posture parameters (θ_F_ and θ_F-H_). The phase of the nonsystematic bias was found to correlate strongly with finger posture, but only weakly with relative finger-head posture. Note that the dashed line represents the results obtained from linear fitting. (**c**) For all six participants, the phase of the nonsystematic bias correlated with the two posture parameters (θ_F_ and θ_F-H_). (**d**) For the sample participant, no correlation exists between the amplitude of the nonsystematic bias and the three posture parameters. (**e**) For all six participants, no correlation exists between the amplitude of the nonsystematic bias and the three posture parameters. **p < 0.01, ***p < 0.001.

Figure 6a shows the nonsystematic bias for all the finger-head posture combinations of the sample participant. When the finger shifts from a vertical position to a horizontal position (θ_F_: from 90° to 0°), the phase of the nonsystematic bias shifts gradually and congruently (θ_p_: from 87° to −13°). However, a change in head posture has no noticeable effect on the phase of the nonsystematic bias. For example, the phase of the nonsystematic bias remains virtually unchanged when the head is moved from the leftward to rightward postures (θ_H_: from 120° to 60°).

An investigation was performed to examine the detailed relations of the finger, head and finger-head postures with the nonsystematic bias across all six participants and the factors affecting the amplitude of the nonsystematic bias (Fig. 6b,c for the phase of the nonsystematic bias, Fig. 6d,e for the amplitude of the nonsystematic bias). Correlation was found between the phase of the nonsystematic bias with both the finger posture (Fig. 6b left, for the sample participant #1: slope = 1.14, R^2^ = 0.93, t = 11.17, p < 0.001, df = 10, data from averaged biases for each finger-head posture. Figure 6c left, for all six participants: slope = 1.08, R^2^ = 0.73, t = 7.76, p < 0.001, df = 22, data from biases averaged across participants) and the finger-head posture (Fig. 6b right, for the sample participant: slope = 0.71, R^2^ = 0.55, t = 3.52, p = 0.006, df = 10, data from averaged biases for each finger-head posture. Figure 6c right, for all six participant: slope = 0.79, R^2^ = 0.72, t = 9.39, p < 0.001, df = 34, data from biases averaged across participants), but not between the phase and the head posture (Fig. 6b middle, for the sample participant: slope = 0.09, R^2^ = 0.003, t = 0.18, p = 0.86, df = 10, data from averaged biases for each finger-head posture. Figure 6c, middle, for all six participants: slope = −0.17, R^2^ = 0.06, t = −1.02, p = 0.32, df = 16, data from biases averaged across participants). The slope of the nonsystematic bias phase vs. the finger posture plot equaled approximately 1 (Fig. 6c, left). In other words, a change in finger posture had a positive and congruent effect on the phase of the nonsystematic bias. However, no correlation was found between any of the three postures and the amplitude of the nonsystematic bias; indicating that the amplitude of the nonsystematic bias is not posture-related (Fig. 6d for [left panel, middle panel, right panel]: slope = [−0.04, −0.06, −0.01], R^2^ = [0.09, 0.09, 0.004], t = [−1.48, −1.50, 0.30], p = [0.15, 0.15, 0.77], df = 22, data from participant #1. Figure 6e, for [left panel, middle panel, right panel]: slope = [0.002, −0.03, −0.012], R^2^ = [0.002, 0.04, 0.02], t = [0.07, −0.86, −0.77], p = [0.94, 0.40, 0.45], df = [22, 16, 34], data from biases averaged across participants). Pairwise correlations across all six participants equaled 0.71 ± 0.11 (R^2^, Mean ± SD) for the phase of the nonsystematic bias (Fig. S2a,b) and 0.15 ± 0.16 for the amplitude of the nonsystematic bias (Fig. S2c,d). Hence, it is inferred that the phase of the nonsystematic bias is consistent across all six participants, but the amplitude of the nonsystematic bias is highly individualized.

Given that the slope of the nonsystematic bias is close to 1 and the phase is similar across participants, this study further examined whether the phase of nonsystematic bias is anchored on the somatotopic reference frame or any other reference frame that is aligned with the skin, such as finger-centered or forearm-centered reference frames. To this end, the results shown in Fig. 6b,c when mapped on the somatotopic reference frame were analyzed (Fig. S3). It was found that the phase is consistent across finger postures when analyzed on the somatotopic reference frames, suggesting that nonsystematic bias is phase-locked to the skin or any other reference frame that is aligned with the skin.

### Systematic vs nonsystematic bias

**Figure 7:**
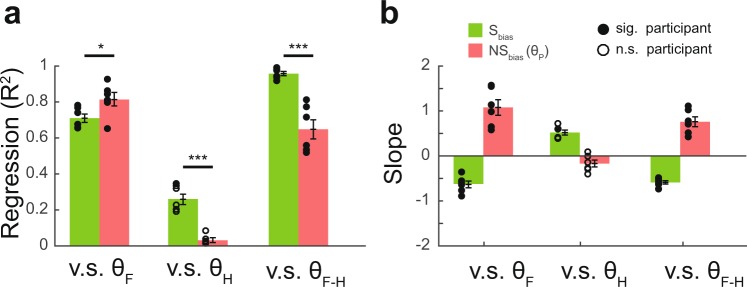
Relationship between systematic bias and phase of nonsystematic bias for six participants. (**a**) Summary of regression results between postures and biases. Each of the finger postures and finger-head postures correlates with the systematic bias and phase of the nonsystematic bias. Head posture correlates weakly with systematic bias, but does not correlate with the phase of nonsystematic bias. (**b**) Slopes of systematic bias and phase of nonsystematic bias vs. head, finger and finger-head posture plots. It is observed that the two biases have opposite slopes in every case. *p < 0.05, ***p < 0.001.

The systematic and nonsystematic bias differ in two main regards. First, the systematic bias is dominated by the combined information of the finger and head postures (Fig. 7a, for v.s. θ_F-H_: Student’s *t* test, t = 5.61, p < 0.001, df = 10, data from regression coefficients of bias v.s. postures of all participants). On the contrary, the phase of the nonsystematic bias is primarily determined by the finger posture (Fig. 7a, for v.s. θ_F_: Student’s *t* test, t = −2.31, p = 0.04, df = 10. Fig. 7a, for v.s. θ_H_: Student’s *t* test, t = 7.13, p < 0.001, df = 10). The head posture correlates only weakly with the systematic bias and has no effect on the phase of the nonsystematic bias. Second, the changes in direction of the bias caused by posture shifts for the systematic bias and the phase of the nonsystematic bias are different. In particular, the slope is negative for the systematic bias and positive for the phase of the nonsystematic bias given changes in the head posture and finger-head posture. Similarly, the slope is positive for the systematic bias and negative for the phase of the nonsystematic bias given changes in the finger posture (Fig. 7b). A further investigation was performed to examine the relationship between the systematic bias and the phase of the nonsystematic bias (Fig. S4). Results showed a consistent negative correlation between the systematic bias and phase of the nonsystematic bias across all six participants; indicating that both biases are modulated by the finger postures.

The sample size of six participants may raise the issue of a lack of statistical power, and it remains unclear whether the aforementioned biases have adequate test-retest reliability. To this end, we performed test-retest experiments that included an additional six participants (Supplementary Text). The results showed that the systematic (Fig. S5) and nonsystematic (Fig. S6) biases were reliable between the test (Fig. S5a,b) and retest (Fig. S6a,b) experiments. Also, the results obtained in these additional six participants were analogous to those observed in the formal experiment (Figs. S5c,d and S6c,d).

Finally, we examined whether the posture-related changes of systematic and nonsystematic biases were simply mediated by the fact that the participants reported the finger or head postures. We showed that it was not the case as participants reported the perceived directions of motion instead of the finger or head postures (Fig. S7).

## Discussion

The present study has revealed plausible mechanisms underlying the reference frame transformation between the somatotopic and allocentric reference frames in response to tactile stimulation. In particular, a consistent deviation in transformation, referred to in this study as a systematic bias, has been found between the veridical and observed directions of the allocentric reference frame. The systematic bias is similar across different participants and is perfectly and linearly predicted by the relative finger-head posture. An additional nonsystematic bias has also been observed: the phase of the nonsystematic bias differs with different stimulus directions and is modulated primarily by the finger posture (i.e., not the head posture). The nonsystematic bias can be well-fitted by a phase-locked cosine function with moment = 2. In other words, an identical nonsystematic bias is induced by tactile stimulations applied in opposite directions. This finding suggests that the nonsystematic bias is associated with inhomogeneous cutaneous senses and is probably involved with the orientation-selective units in the S1 cortex^55^. Furthermore, given a change in the finger or head orientation induced dramatically different changes in the systematic and nonsystematic biases, it is inferred that the two biases are mediated by distinctively different mechanisms.

The present results have shown that the systematic bias is linearly predicted by the relative finger-to-head posture; indicating the importance of both the finger posture and the head posture in determining the bias. These findings are reminiscent of the observations of Carter *et al*.^56^, who found that the eye position affected the perceived tactile direction, and those of Volcic *et al*.^57^, who found that the head posture affected the perceived direction in parallelity tests. Tactile orientation was originally hypothesized to be perfectly encoded in an allocentric reference frame. However, this notion was challenged by the data collected by Hammerschmidt^18^. Moreover, in recent years, parallelity and mental rotation experiments have shown that the perceived orientation cannot be explained by any single reference frame when performed on the horizontal^45,47^, midsagittal^47^, or frontoparallel planes^48^. However, a reference frame that is an intermediate of multiple frames can account for such tactile orientation bias^49–51^. Many different sources may contribute to the reference frame, including the position or posture of the skin^16^, hand^19,58^, arm^59–61^, and body^62^. These findings are analogous to those obtained in studies on motor-sensory coordination^59–61^, which show that a hybrid frame of reference is constructed to combine parallel multisensory information. In general, these previous findings imply a multisensory nature of tactile perception. That is, tactile information needs to be mapped onto other reference frames such that the somatosensory, visual, auditory and motor functions can be integrated as a single holistic system^16,56–64^.

The phase of the nonsystematic bias between the reported tactile direction and the veridical direction is anchored on the somatotopic reference frame (i.e., not the head posture). To the best of our knowledge, the nonsystematic bias of human touch has never been reported. However, the finding that nonsystematic bias can be fitted by a cosine function with a moment of two is reminiscent of the theory of tactile anisotropy (also known as the oblique effect), which states that tactile acuity tends to be better at certain orientations^62,65,66^. Neuronal data provide plausible support for the origin of the nonsystematic bias^55^. Specifically, some neurons in the primary somatosensory cortex are highly selective for the orientation or direction of scanning gratings^55,67^. In other words, transformation of the reference frames may be mediated by these orientation-selective units, in which a high percentage of neurons prefer the proximal-distal orientation^68^. Another possible explanation for the origin of the nonsystematic bias may be inhomogeneous finger compliance or inhomogeneous receptor properties^69^ in response to motion stimulus presented to the finger. However, all these suggestions seem possible because the nonsystematic bias is constant when calculating the bias in the somatotopic reference frame^62,65,66,68,69^.

The evidence presented in this study suggests that systematic and nonsystematic biases reflect two completely different properties underlying the transformation from the somatotopic frame to the other reference frames. In particular, systematic bias is determined primarily by the relative finger-head posture; reflecting its multisensory nature. By contrast, nonsystematic bias is determined only by the finger posture, indicating most probably that it has a somatosensory nature. Furthermore, the slope of the phase of nonsystematic bias is close to 1 for most of the participants, suggesting that nonsystematic bias is phase-locked to the skin or any other reference frame that is aligned with the skin, such as finger-centered and forearm-centered reference frames. The present findings are inconsistent with the hypothesis that all sensory modalities are remapped to a common frame of reference^70–72^. In fact, neurons in the posterior parietal cortex, such as the ventral intraparietal (VIP) area, apply a variety of reference frames, including intermediate somatosensory and visual reference frames, to encode the stimulus location^73^, indicating that the integration between touch and vision is mediated by a coexistence of multiple reference frames.

The formal experiment included only six participants, a sample size that might be susceptible to type II errors. Also, this small sample size could limit our ability to analyze the variance across participants. Except for that, the experimental design adopted in this study has several important advantages. First, it enables the linearity of change in bias phase to be examined as a function of posture; a property that parallels the gradual shift of the receptive field in multisensory neurons observed in the ventral intraparietal area^73,74^, lateral intraparietal area^75^, ventral premotor area^76^, and superior colliculus^70,71^. Second, the present study on the perceived direction of stimulus motion has ecological value as haptics usually involves motion between the finger and the object^6,77^. However, tactile flow, i.e., the motion information obtained by the finger, is still required for subsequent motor planning. Finally, the tactile stimulation applied in this study is implemented with a directional precision of 1° and an indentation depth precision of 1 μm^54^. As a result, it provides the means to extract bias patterns with relatively small magnitudes, such as the nonsystematic bias reported herein.

## Methods

### Participants

A total of six participants (four males, two females, 26 to 35 years of age) participated in the formal experiment. An additional eight participants (six males, two females, 20 to 36 years of age) participated in the test-retest experiments (Supplementary Text). The protocol was approved by the Institutional Review Board of Human Research of Chang Gung Medical Foundation and written informed consent was obtained from all participants. All methods were performed in accordance with the regulations of Human Subjects Research Act in Taiwan and with the guidelines of the Declaration of Helsinki, 1975.

### Tactile stimulator

The ball measured 20 mm in diameter and was engraved with square-wave gratings with a depth of 500 μm, a wavelength of 4.0 mm and a 45% duty cycle (Fig. 1a). A three-motor controller was employed to control the tactile stimulus (Fig. 1b) so that the direction of motion and indentation depth could be precisely controlled (see Pei *et al*. 2014 for details). During the experiment, white noise was played through an earphone to prevent the participant from hearing the motor noise.

### Experimental set-up

Each participant sat in front of a table with the left upper arm and forearm held by arm holders to maintain the arm position (Fig. 1c). The angle of the elbow joint was not measured but was kept constant across all the experiments. Specifically, for the left upper limb, the participant’s index finger and wrist were kept at neutral position (straight), forearm supinated at 90°, and elbow flexed at 90°. The finger orientation was adjusted by changing the participant’s shoulder abduction and internal rotation postures. In order to make sure that these postures were kept stationary during each session of the experiment; a forearm holder was utilized to support the wrist and an arm holder was employed to support the elbow. For the head posture, a pad was placed lateral to the head and the participant needs to tilt the head to touch the pad so that the head posture can be maintained.

The stimulus ball was placed immediately in front of the left index finger. In addition, a video display screen was placed between the participant and the tactile stimulator to provide experimental instructions and to enable the participant to report the perceived direction of motion of the ball. In setting up the experimental process, from posterior to anterior with respect to the participant’s head, the eyes, video display, tactile ball, and left index fingerpad were perfectly aligned along the posterior-to-anterior axis. The allocentric reference frame (coordinate) was defined on the frontoparallel plane (Fig. 1c).

### Experimental design of motion stimulation

For each participant, the finger, forearm, and head holders were adjusted to ensure between-participant consistency of finger and head postures. For the intensity of tactile stimulation, tactile motion was presented by the miniature tactile motion stimulator^54^ (Fig. 1a,b) with indentation depth of 1000 μm, and had the indentation rate of 2 mm/s which was far beyond the sensation threshold of indentation rate (>0.3 mm/s)^78^. In this setup, the discriminability of stimulus direction^54^ was 11.4° ± 2.5°. Tactile motion was presented with a speed of 40 mm/s using a square wave grating ball with a wavelength of 2 mm^79^ and temporal frequency of 10 Hz^80^, both of which were beyond the threshold of tactile perception^13^.

At the beginning of each trial, the participant visually fixated on a cross presented at the center of the video display. After 1 second fixation, the rotating ball was indented on the index fingertip with indentation depth of 1 mm. Tactile stimulation was then applied for 1 second in one of the 24 different directions (0° to 345° in 15° increments) at a speed of 40 mm/s. The ball was then withdrawn from the fingerpad (Fig. 2a). After the ball was moved away, the participant reported the perceived direction of motion (${R}_{i}$) using a mouse to click on an appropriate point on a circle shown on the video display. After that, a gray blank appeared on the screen for 1 second (Fig. 2a left). After the participant clicked on the circle, the screen was presented in gray blank for a period of 1 second and then shown again for the following trial.

The stimulation trials were performed in accordance with a 3-by-4 factorial finger-and-head posture combination design consisting of three head postures and four finger postures. In particular, the finger postures (θ_F_) were set as 90°, 60°, 30° or 0°, while the head postures (θ_H_) were set as 120°, 90° or 60°; yielding a total of 12 different posture combinations (Fig. 2b). Note that parameters θ_H_ and θ_F_ were both defined on the allocentric reference frame. A total of 96 trials were performed for each posture combination (24 directions * 2 times * 2 blocks).

### Definition of bias

The perceptual bias (${B}_{i}$) was quantified as the difference between the reported stimulus direction (${R}_{i}$) and the veridical direction (${V}_{i}$) on the allocentric reference frame (${B}_{i}$ = ${R}_{i}$– ${V}_{i}$). For each posture combination, the systematic bias, $S$, was computed as the mean of the perceptual biases across all 24 directions, i.e., $S=\frac{1}{24}{\sum }_{i=1}^{24}{B}_{i}$. In addition, the nonsystematic bias, $N{S}_{i},$ for the veridical direction was computed as the difference between the perceptual bias and the systematic bias, i.e., $N{S}_{i}={B}_{i}-S$.

### Fitting for nonsystematic bias

The nonsystematic bias was fitted as a function of the veridical direction using a cosine function with moment = 2 (Fig. S1) that yields two full oscillations in one complete direction cycle, i.e., $N{S}_{i}=A\,\cos (2{V}_{i}-2{\theta }_{p}),$ where ${\rm{A}}$ and ${{\rm{\theta }}}_{{\rm{p}}}$ represent the amplitude and phase corresponding to the maximal nonsystematic bias and are free parameters determined using the least-squares fitting method.

### Statistical analysis

The perceptual bias, the difference between the perceived and veridical directions on the allocentric reference frame, was first computed for each trial. For each participant, the perceptual bias for each direction of motion was the circular mean of perceptual bias across the four repetitions. The systematic bias for each posture was the mean across stimulus directions (Fig. 3). The amplitude (A) and phase (θ_p_) of the nonsystematic bias were retrieved from cosine fit to the nonsystematic bias at its peak position. To evaluate the relationship between postures and biases, we applied Pearson’s correlation (simple regression model) to compute the correlation between posture angles to the parameters of systematic (Fig. 4) or nonsystematic bias (Fig. 6). For each of the three posture conditions, θ_F_, θ_H_, and θ_F-H_, we applied Student’s *t* test to compare the values of regression coefficients (R^2^) between systematic and nonsystematic biases to evaluate the degree to which the two biases was modulated by the postures (Fig. 7). To examine whether there was inconsistency of goodness-of-fit (R^2^) of cosine fit across postures, we applied repeated-measures ANOVA for each of the finger posture (θ_F_), head (θ_H_), and finger-head postures (θ_F-H_) (Fig. S1b). We used Pearson’s correlation to examine whether nonsystematic bias is a function of finger posture on the allocentric reference frame (Fig. S3), and, finally, to evaluate the relationship between systematic bias and the phase of nonsystematic bias for each participant (Fig. S4).

## Supplementary information

Supplementary information.
