# Author Correction: Relative posture between head and finger determines perceived tactile direction of motion

**DOI:** 10.1038/s41598-020-65967-1

**Published:** 2020-06-04

**Authors:** Yueh-Peng Chen, Chun-I. Yeh, Tsung-Chi Lee, Jian-Jia Huang, Yu-Cheng Pei

**Affiliations:** 1 Department of Physical Medicine and Rehabilitation, Chang Gung Memorial Hospital, Taoyuan, Taiwan; 2 Center of Vascularized Tissue Allograft, Chang Gung Memorial Hospital at Linkou, Taoyuan, Taiwan; 3 School of Medicine, Chang Gung University, Taoyuan, Taiwan; 4 Healthy Aging Research Center, Chang Gung University, Taoyuan, Taiwan; 5 Center for Artificial Intelligence in Medicine, Chang Gung Memorial Hospital at Linkou, Taoyuan, Taiwan; 6 Department of Psychology, National Taiwan University, Taipei, Taiwan

Correction to: *Scientific Reports* 10.1038/s41598-020-62327-x, published online 26 March 2020

This Article contains errors in the Results section.

Under the subheading ‘Systematic Bias’,

“Interestingly, the systematic bias was approximately zero for postures of θ_H_ = 120° and θ_F_ = 90°, θ_H_ = 90° and θ_F_ = 60°, and θ_H_ = 60° and θ_F_ = 30°, i.e., postures under which the difference between the finger and head angles was −30° (θ_H_ − θ_F_ = −30°).”

should read:

“Interestingly, the systematic bias was approximately zero for postures of θ_H_ = 120° and θ_F_ = 90°, θ_H_ = 90° and θ_F_ = 60°, and θ_H_ = 60° and θ_F_ = 30°, i.e., postures under which the difference between the finger and head angles was −30° (θ_F_ − θ_H_ = −30°).”

Under the subheading ‘Nonsystematic Bias’,

“However, no correlation was found between any of the three postures and the amplitude of the nonsystematic bias; indicating that the amplitude of the nonsystematic bias is not posture-related (Fig. 6d for [left panel, middle panel, right panel]: slope = [−0.04, −0.06, −0.01], R^2^ = [0.09, 0.09, 0.004], t = [−1.48, −1.50, 0.30], p = [0.15, 0.15, 0.77], df = 22, data from participant #1.”

should read:

“However, no correlation was found between any of the three postures and the amplitude of the nonsystematic bias; indicating that the amplitude of the nonsystematic bias is not posture-related (Fig. 6d for [left panel, middle panel, right panel]: slope = [−0.04, −0.06, 0.01], R^2^ = [0.09, 0.09, 0.004], t = [−1.48, −1.50, 0.30], p = [0.15, 0.15, 0.77], df = 22, data from participant #1.”

Under the subheading ‘Systematic vs nonsystematic bias’,

“In particular, the slope is negative for the systematic bias and positive for the phase of the nonsystematic bias given changes in the head posture and finger-head posture. Similarly, the slope is positive for the systematic bias and negative for the phase of the nonsystematic bias given changes in the finger posture (Fig. 7b).”

should read:

“In particular, the slope is negative for the systematic bias and positive for the phase of the nonsystematic bias given changes in the finger posture and finger-head posture. Similarly, the slope is positive for the systematic bias and nonsignificant for the phase of the nonsystematic bias given changes in the head posture (Fig. 7b).”

