# Supplementary material for: Relative posture between head and finger determines perceived tactile direction of motion
Source: Sci Rep. 2020 Mar 26;10:5494. doi: 10.1038/s41598-020-62327-x (PMC7099024; doi:10.1038/s41598-020-62327-x)
Supplement: Supplementary file 1 — Supplementary information. [file 41598_2020_62327_MOESM1_ESM.docx]

**Supplementary Information**

**Article in *Scientific Reports***

**Relative posture between head and finger determines perceived tactile direction of motion**

Yueh-Peng Chen, PhD; Chun-I Yeh, PhD; Tsung-Chi Lee, MS; Jian-Jia Huang, PhD; Yu-Cheng Pei, MD, PhD*

*Correspondence: Yu-Cheng Pei, MD, PhD, Department of Physical Medicine and Rehabilitation, Chang Gung Memorial Hospital, No. 5 Fushing St., Taoyuan 333, Taiwan. E-mail: [yspeii@gmail.com](mailto:yspeii@gmail.com)

**Supplementary Text**

**Test-retest reliability experiment**

The formal experiment included only six participants, a sample size that might be susceptible to type II errors. Also, this small sample size could limit our ability to analyze the variance across participants. To further tackle these questions, we explored the test-retest reliability by recruiting an additional eight participants (six males and two females, 20 to 36 years of age).

In this test-retest experiment, we chose three finger and head postures ([θ_F_, θ_H_] = [90°, 60°], [60°, 90°], and [30°, 120°]) instead of the original 12 posture combinations to save time and reduce the burden on participants. The three posture combinations covered from positive to negative systematic biases that are more balanced than the biases from the other posture combinations. Each participant received the test-retest experiments which were performed one week apart to examine the test-retest reliability.

Among the eight participants, two were excluded as the circular standard deviation of the systematic bias was higher than 20°. Supplementary Figures S5a, b – left and right panels illustrate the systematic bias in the test and retest (follow-up) experiments, respectively. The systematic bias in the test-retest experiments was quite analogous with that in the formal experiment in the main text (Fig. S5c). Comparisons among the formal experiment and the test-retest experiments showed that the averages of the systematic bias were similar in the reliability experiment (test vs. retest), but were slightly different from those observed in the formal experiment (Fig. S5d).

The phase of the nonsystematic bias (θ_P_) also showed similar test-retest values in the peak bias in each of six participants and in the mean among participants (Fig. S6a, b). Again, the phase of the nonsystematic bias was analogous with that observed in the formal experiments (Fig. S6c, d). To sum up, these data support adequate reliability in this experimental setup by showing good test-retest reliability and similar results across participants both in the systematic and the nonsystematic biases.

**Supplementary Figures**


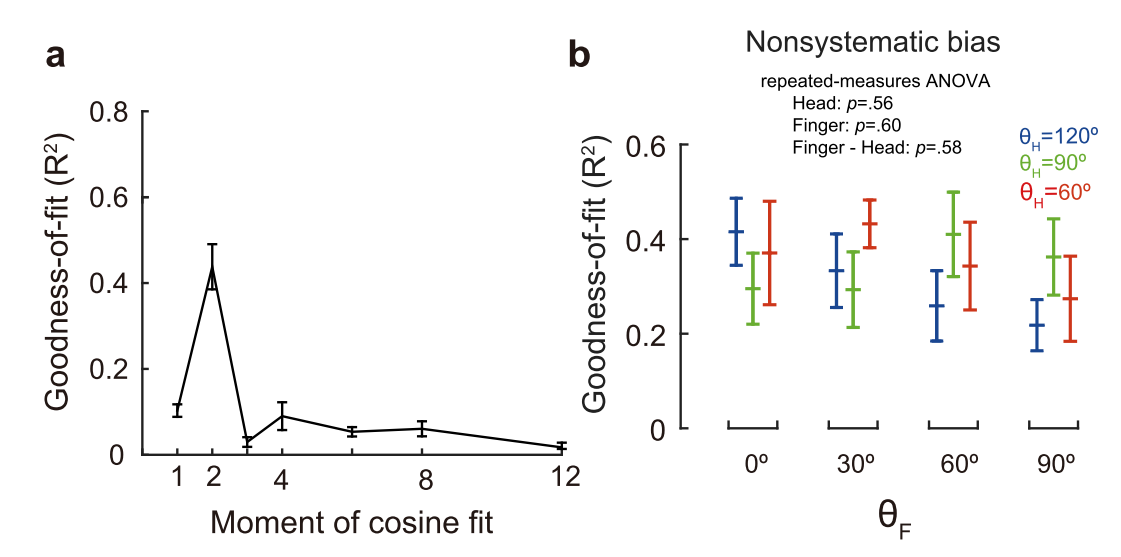


**Supplementary Figure S1.** Performance of cosine fit for all posture combinations. (**a**) For the cosine fit, the goodness-of-fit peaks for moment = 2. (**b**) The goodness-of-fit is comparable across all posture combinations. Analyses were performed with one-way repeated-measures ANOVA. For head postures, F(2, 42) = .59, p = .56, for finger postures, F(3, 45) = .63, p = .60, for finger-head postures, F(11, 44) = .87, p = .58. Error bars represent S.E.M.


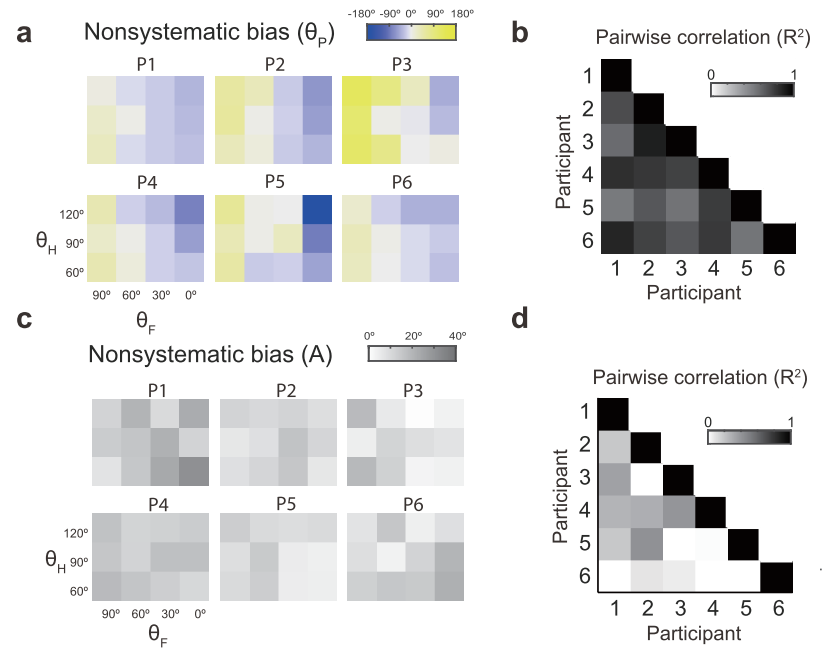


**Supplementary Figure S2.** Nonsystematic bias of each participant and pairwise correlations of bias between participants. (**a**) For each participant, the phase of nonsystematic bias is color-coded across 12 posture combinations. (**b**) Pairwise correlations of the phase of nonsystematic bias for six participants. (**c**) Color-coded amplitudes of nonsystematic bias for each participant. (**d**) Pairwise correlations of the amplitude of the nonsystematic bias for six participants.


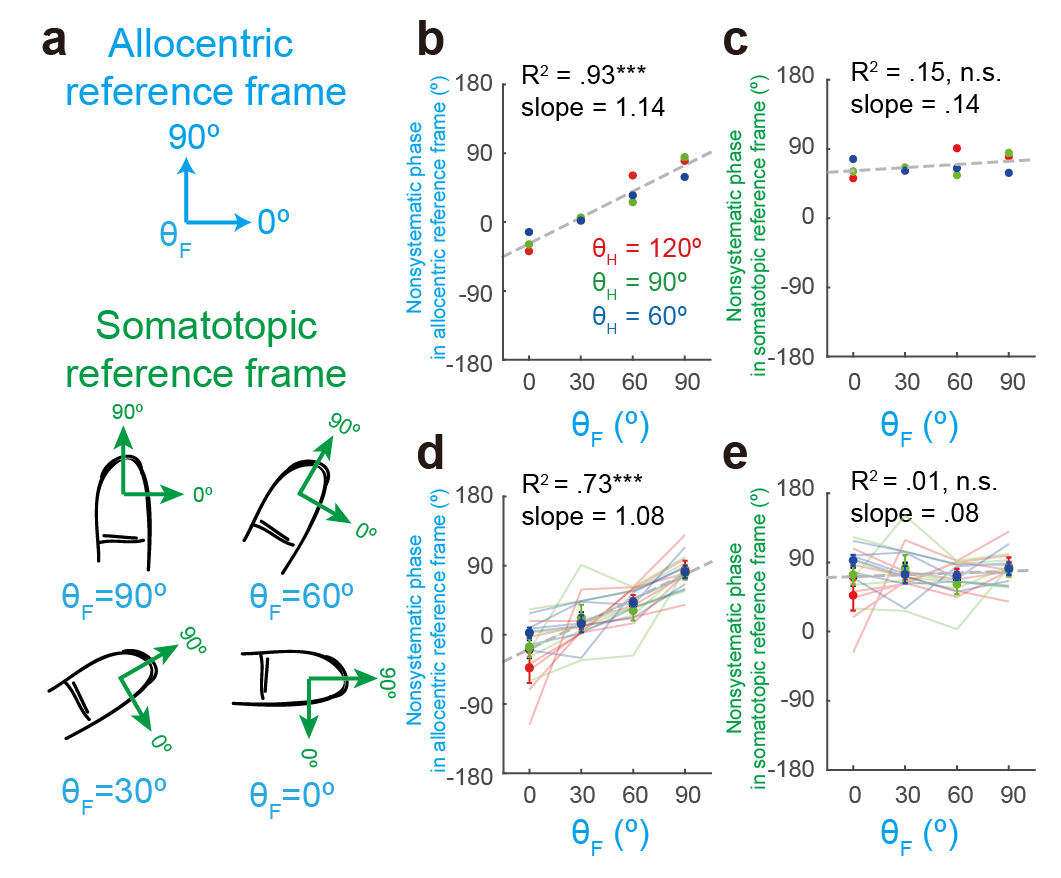


**Supplementary Figure S3.** The phase of nonsystematic bias as function of finger posture when analyzed in the allocentric or somatotopic reference frame. (**a**) The allocentric reference frame was marked in blue and the somatotopic reference frame in green. (**b**) The phase of nonsystematic bias of a sample participant correlated with finger posture when corresponding to the allocentric reference frame. For the sample participant: slope = 1.14, R^2^ = .93, t = 11.17, p < .001, df = 10, data from averaged biases for each finger-head posture. (**c**) For the sample participant, the phase of nonsystematic bias correlated weakly with finger posture when corresponding to the somatotopic reference frame. Slope = .14, R^2^ = .15, t = 1.35, p = .21, df = 10, data from averaged biases for each finger-head posture. (**d**) The phase of nonsystematic bias correlated with finger posture for all six participants. For all six participants: slope = 1.08, R^2^ = .73, t = 7.76, p < .001, df = 22, data from biases averaged across participants. (**e**) For the six participants, the phase of nonsystematic bias did not correlate with finger posture when corresponding to the somatotopic reference frame. Slope = .08, R^2^ = .01, t = .56, p = .58, df = 22, data from biases averaged across participants. * p < .05, *** p < .001.

**
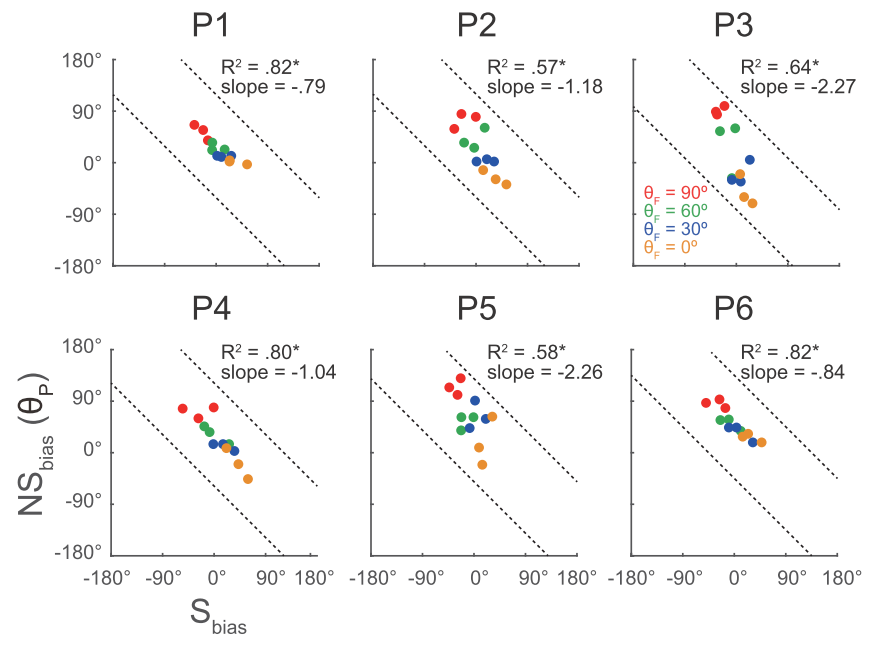
**

**Supplementary Figure S4.** Systematic bias as function of phase of nonsystematic bias for six participants. A significant negative correlation is found between the biases for all six participants because both biases are modulated by finger posture. For participants P1~P6: slope = [-.79, -1.18, -2.27, -1.04, -2.26, -.84], R^2^ = [.82, .57, .58, .80, .64, .82], t = [-6.81, -3.63, -3.70, -6.25, -4.23, -6.79], p = [< .001, .005, .004, < .001, .002, <.001], df = 10. * p < .05.


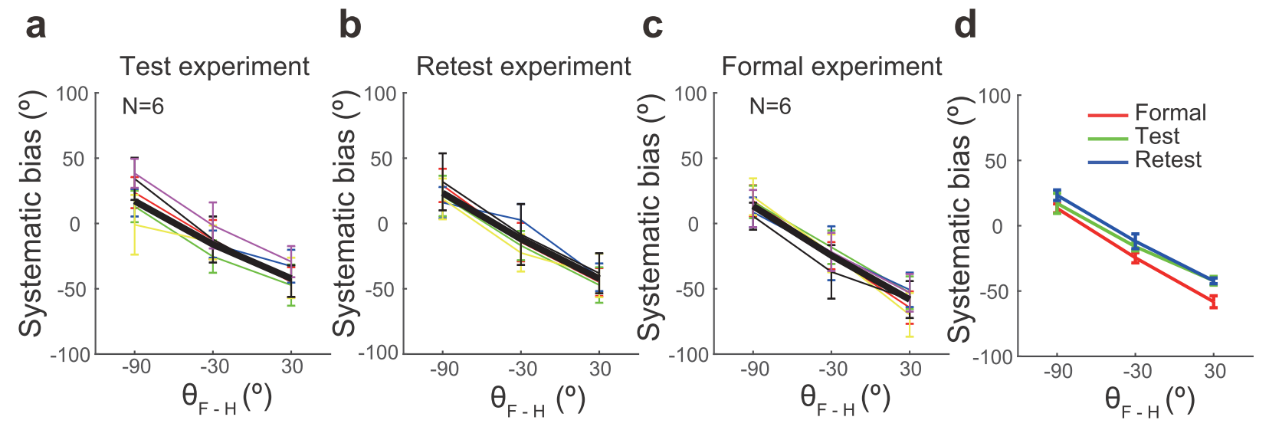


**Supplementary Figure S5.** Systematic bias observed in the reliability experiment. (**a**) Three finger and head postures ([θ_F_, θ_H_] = [90°, 60°], [60°, 90°], and [30°, 120°]) were chosen for the reliability experiment. Among eight participants, six were included as their circular standard deviation of systematic bias was smaller than 20°. Each solid colored line represents the data from individual participant and the black line represents the mean averaged value across the six participants. (**b**) Retest experiment for those six participants after one week. (**c**) Results obtained from three of formal finger and head postures. (**d**) Test-retest experiments were more similar with each other but their values were slightly different from those in the formal experiment.


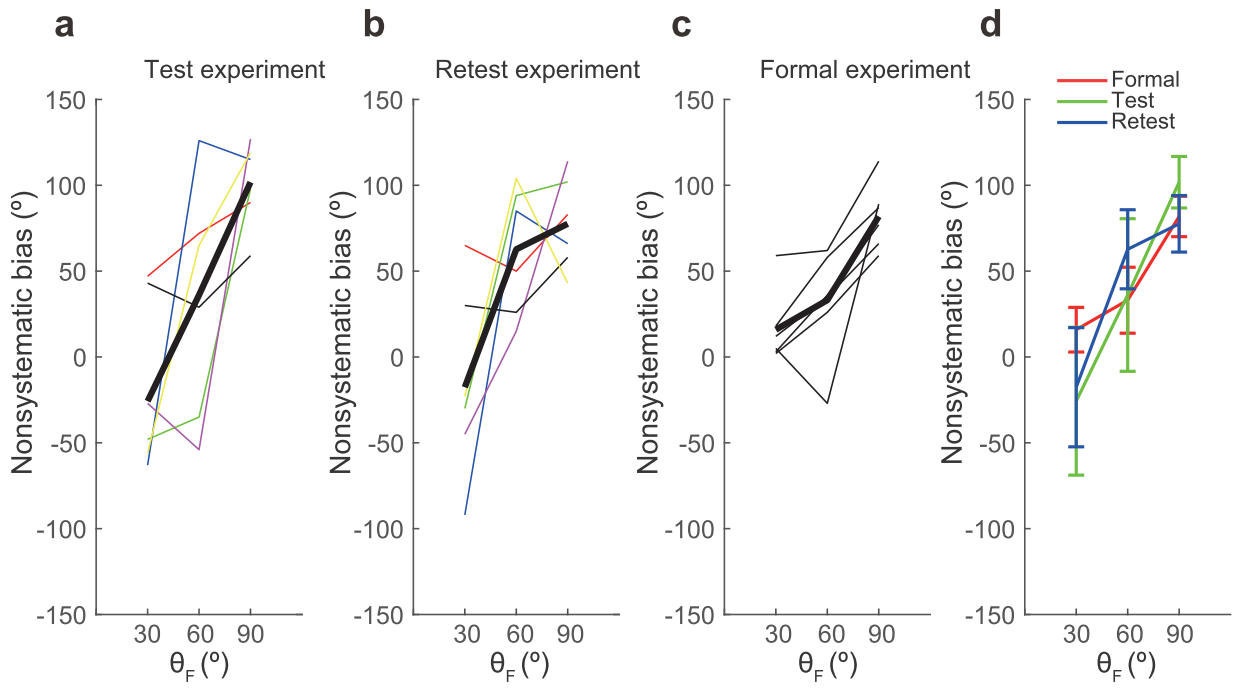


**Supplementary Figure S6.** Phase of nonsystematic bias (θ_P_) in the reliability experiment (**a, b**) The six participants showed similar phase of nonsystematic bias in both test (**a**) and retest (**b**) experiments. (**c**) Phase of nonsystematic bias in the formal experiment. (**d**) The mean nonsystematic bias in the reliability experiment across participants in the test, retest and formal experiments are similar under the same conditions.


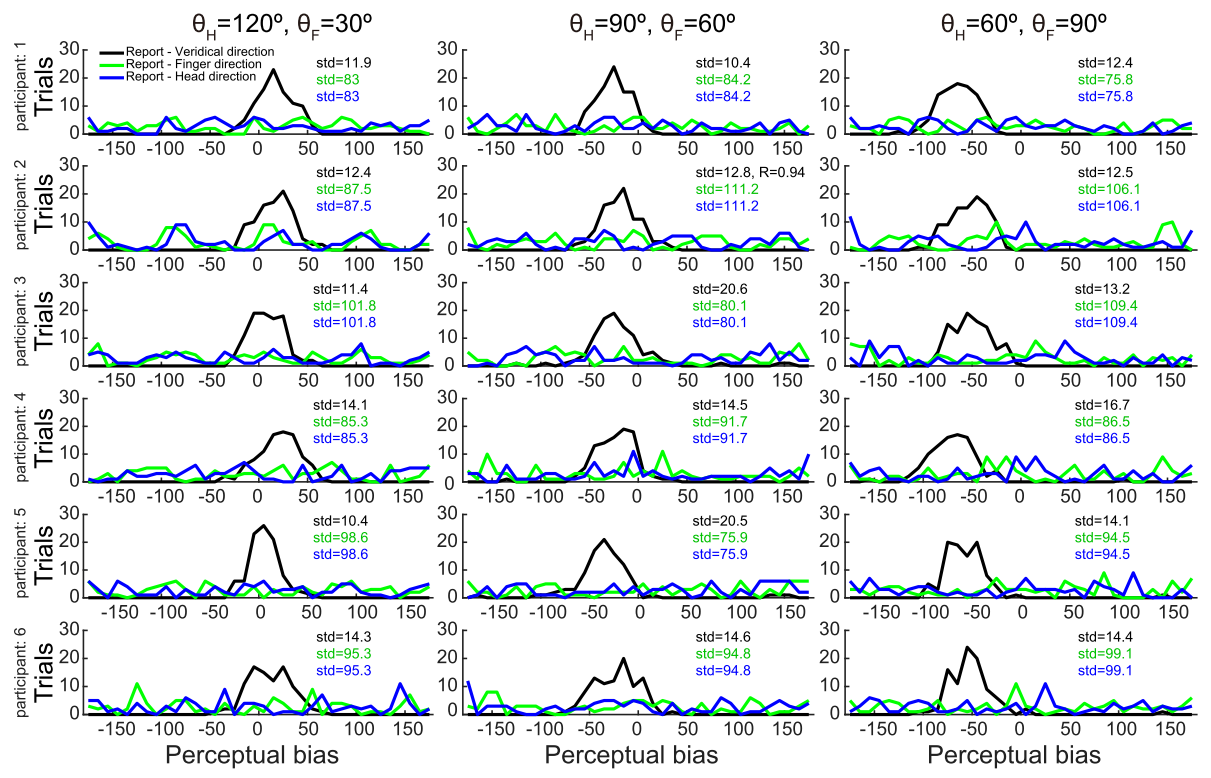


**Supplementary Figure S7.** Histogram of perceptual bias for each of the six participants. Three types of perceptual bias were reported, including (1) reported direction – veridical direction (black line), (2) reported direction – finger direction (green line), (3) reported direction - head direction (blue line). The columns of subplots represent the three head and finger postures ([θ_H_, θ_F_] = [120°, 30°], [90°, 60°], [60°, 90°]), and the row of subplots represents the six participants. Circular standard deviation (std) was computed for each subplot.
